# Predictive factors associated with liver fibrosis and steatosis by transient elastography in patients with HIV mono‐infection under long‐term combined antiretroviral therapy

**DOI:** 10.1002/jia2.25201

**Published:** 2018-11-05

**Authors:** Hugo Perazzo, Sandra W Cardoso, Carolyn Yanavich, Estevão P Nunes, Michelle Morata, Nathalia Gorni, Paula Simplicio da Silva, Claudia Cardoso, Cristiane Almeida, Paula Luz, Valdilea G Veloso, Beatriz Grinsztejn

**Affiliations:** ^1^ Laboratory of Clinical Research in STI/AIDS (LAPCLIN‐AIDS) National Institute of Infectious Diseases Evandro Chagas‐Oswaldo Cruz Foundation (INI/FIOCRUZ) Rio de Janeiro Brazil; ^2^ Plataform of Clinical Research National Institute of Infectious Diseases Evandro Chagas‐Oswaldo Cruz Foundation (INI/FIOCRUZ) Rio de Janeiro Brazil; ^3^ Department of Nutrition National Institute of Infectious Diseases Evandro Chagas‐Oswaldo Cruz Foundation (INI/FIOCRUZ) Rio de Janeiro Brazil

**Keywords:** liver disease, hepatic fibrosis, fatty liver, HIV infection, HIV care continuum

## Abstract

**Introduction:**

Non‐alcoholic fatty liver disease is characterized by the presence of hepatic steatosis and can be associated with fibrosis progression, development of cirrhosis and liver‐related complications. Data on the prevalence of liver fibrosis and steatosis in HIV patients remain contradictory in resource‐limited settings. We aimed to describe the prevalence and factors associated with liver fibrosis and steatosis in patients with HIV mono‐infection under long‐term antiretroviral therapy (ART) in Rio de Janeiro, Brazil.

**Methods:**

Clinical assessment, fasting blood collection and liver stiffness measurement (LSM)/controlled attenuation parameter (CAP) by transient elastography were performed on the same day for this cross‐sectional study (PROSPEC‐HIV study; NCT02542020). Patients with viral hepatitis co‐infection, ART‐naïve or missing data were excluded. Liver fibrosis and steatosis were defined by LSM ≥ 8.0 kPa and CAP ≥ 248 dB/m respectively. HIV history, cumulative and current ART regimens were evaluated. Multivariate logistic regression models adjusted for age and gender were performed.

**Results:**

In total, 395 patients (60% female; median age of 45 (IQR, 35 to 52) years, body mass index = 25.7 (23.2 to 29.4) kg/m^2^, alanine aminotransferase = 30 (23 to 42) IU/L, duration of ART for 7 (4 to 14) years) were included. LSM and CAP were reliable in 93% (n = 367) and 87% (n = 344) respectively. The prevalence of fibrosis and steatosis were 9% (95% confidence interval (CI), 7 to 13) and 35% (95% CI, 30 to 40) respectively. The following factors were associated with fibrosis (odds ratio (OR) (95% CI)): older age (per 10 years; 1.80 (1.27 to 2.55); *p* = 0.001) and CD4+ count <200 cells/mm^3^ (7.80 (2.09 to 29.09), *p* = 0.002). Type 2 diabetes had a trend towards the presence of liver fibrosis (2.67 (0.96 to 7.46), *p* = 0.061). Central obesity (10.74 (4.40 to 26.20), *p* < 0.001), type 2 diabetes (9.74 (3.15 to 30.10), *p* < 0.001), dyslipidaemia (2.61 (1.35 to 5.05), *p* = 0.003) and metabolic syndrome (4.28 (2.45 to 7.46), *p* < 0.001) were associated with steatosis. A dominant backbone ART regimen of zidovudine (AZT), d4T, ddI or ddC was associated with steatosis (1.90 (1.07 to 3.38), *p* = 0.028) independently of metabolic features.

**Conclusion:**

Integrated strategies for preventing non‐communicable diseases in people with HIV mono‐infection are necessary to decrease the burden of liver diseases.

Clinical Trial Number: NCT02542020.

## 1 Introduction

Co‐infection with viral hepatitis is the leading cause of liver‐related complications in people living with HIV infection 1. However, the increased burden of non‐alcoholic fatty liver disease (NAFLD) in patients with HIV infection cannot be ignored 2. The clinical presentation of NAFLD can range from simple steatosis to non‐alcoholic steatohepatitis (NASH) and its associated complications 3. Liver biopsy has long been considered the gold standard for both diagnosing steatosis and fibrosis staging; however, this invasive method has potential for complications, and is not widely available 4. Non‐invasive methods might be used to diagnosis and to evaluate NAFLD/NASH as an alternative to liver biopsy 5. Transient elastography has been validated as a non‐invasive method to assess liver fibrosis by liver stiffness measurement (LSM) 6 and steatosis by controlled attenuation parameter (CAP) 7.

In the general population, metabolic factors are likely contributors to the current NAFLD epidemic 8. People with obesity and type 2 diabetes are at high risk for the development of NAFLD and NASH 9. In people with HIV infection, few studies have reported the burden of liver fibrosis and steatosis 10, 11, 12, 13, 14, 15. However, in resource‐limited settings, prevalence data remain scarce while risk factors associated with hepatic fibrosis and steatosis remain poorly defined in patients with HIV mono‐infection. Current guidelines do not include routine NAFLD screening due to cost and uncertainties about the clinical benefits 9. However, identification of NAFLD can be used to stratify individuals with high risk for bridging fibrosis or cirrhosis. In people with HIV mono‐infection, further studies are needed to evaluate the impact of long‐term antiretroviral therapy (ART), as well as the identification of different pathways for development of liver fibrosis and steatosis. We aimed to describe both the prevalence and risk factors associated with liver fibrosis and steatosis in a cohort of patients with HIV mono‐infection under long‐term ART followed in Rio de Janeiro, Brazil.

## 2 Methods

### 2.1 Study population

This cross‐sectional analysis of baseline data from the PROSPEC‐HIV study (NCT02542020) included adults with HIV infection on ART for more than six months from June 2015 to March 2017. Patients co‐infected with viral hepatitis, defined as having either a positive HCV antibody or positive HBsAg test result, were excluded. Clinical assessment, blood sample collections and transient elastography (TE) were performed on the same day. The study protocol was approved by the Ethics Committee from INI/FIOCRUZ (IRB 32889514.4.0000.5262) and all participants signed an informed consent prior to enrolment in the PROSPEC‐HIV study.

### 2.2 Clinical evaluation and blood tests

Clinical records included age, gender, self‐reported race, years of study, anthropometric measures, blood pressure (Omron Healthcare, Kyoto, Japan), alcohol consumption (quantified by the Alcohol Use Disorders Identification Test (AUDIT) score and drinking patterns), smoking (never, past or current), presence of co‐morbidities and use of concomitant medications. The presence of central obesity, dyslipidaemia, blood hypertension, type 2 diabetes and metabolic syndrome were defined according to the International Diabetes Federation 16. Patients with an AUDIT score ≥8 points were categorized as hazardous drinkers 17. Blood tests were performed after an overnight fasting and analysed in a central laboratory using a Dimension‐RxL‐Max (Siemens Healthcare Diagnostic, Deerfield, IL, USA) analyzer. Serum alanine aminotransferase (ALT), aspartate aminotransferase (AST) and gamma‐glutamyltransferase (GGT) were measured using an enzymatic assay. The upper limit of normal (ULN) minotransferases values were 78 IU/L and 37 IU/L for ALT and AST respectively. Plasma levels of total and high‐density lipoprotein (HDL)‐cholesterol and triglycerides were determined using enzymatic methods. Low‐density lipoprotein (LDL)‐cholesterol levels were calculated using the Friedewald equation and glucose was measured using the hexokinase method. Fibrosis‐4 score (FIB‐4), Aspartate‐to‐Platelet Ratio Index (APRI) and NAFLD fibrosis score (NFS), predictive models for detection of fibrosis that use simple laboratorial parameters, were calculated.

### 2.3 Transient elastography

Transient elastography was performed by a single experienced (>2000 examinations) operator (HP) following a validated procedure using an M probe of FibroScan (EchoSens, Paris, France) in patients after an overnight fasting 18. Briefly, the probe was placed in an intercostal space at the level of the right hepatic lobe and the operator, assisted by a time‐motion ultrasound image, located a liver portion free of large vascular structures where measures were acquired. Final results were expressed as a median of 10 valid measures. Transient elastography was considered reliable when the following criteria had been met: (i) 10 successful measurements; (ii) an interquartile range (IQR) lower than 30% of the median value of LSM for fibrosis and CAP for steatosis; and (iii) a success rate of more than 60% 19. LSM or CAP failure was defined by the absence of valid measurements during TE exam. The presence of liver fibrosis and steatosis were defined by LSM ≥ 8.0 kPa 20 and CAP ≥ 248 dB/m 7 respectively. Participants with diagnosis of liver fibrosis and/or steatosis were referred for follow‐up by specialists in our Institution.

### 2.4 HIV infection and c‐ART history

Since 1990, INI/FIOCRUZ, a national reference centre for HIV care, research and training, has maintained an electronic longitudinal clinical database of people living with HIV. Cohort data are regularly updated by trained investigators using medical records, laboratory results and pharmacy dispensing records, which include ART prescription details 21. The following data are available for the INI/FIOCRUZ HIV cohort: (i) date of first positive HIV antibody test; (ii) information on ART regimens (drugs, initiation and discontinuation dates, and dosage); (iii) CD4+ T‐lymphocyte count and HIV viral load; and (iv) viral hepatitis serology. The duration of HIV infection and duration of ART were defined by subtracting the date of inclusion in the study from the date of first positive HIV antibody and date of initiation of first antiretroviral drug respectively. Results from the most recent CD4+ T‐lymphocyte count and HIV‐1 RNA test obtained prior to or after study inclusion were used in the analysis. Antiretroviral regimens were classified as backbone drug (tenofovir (TDF) vs. zidovudine (AZT)) and core drug classes (non‐nucleoside reverse‐transcriptase inhibitors (NNRTIs) vs. protease inhibitor (PIs) or integrase strand transfer inhibitors (INSTIs)). The use of TDF, abacavir, emtricitabine or tenofovir alafenamide were considered as a TDF‐backbone. Similarly, the use of AZT, didanosine (ddI), stavudine (d4T) or zalcitabine (ddC) were considered as an AZT‐backbone. Treatment by any NNRTI or PI/INSTI defined these core drug classes. Backbone and core drug classes for current ART were defined based on the antiretroviral regimen used at study entry. Backbone and core drug classes for cumulative ART exposure were defined by the sum of years of drugs use; the “most used” backbone and core drugs were those with the highest cumulative exposure.

### 2.5 Statistical analysis

Categorical variables were reported as absolute (n) and relative frequencies (%) and continuous variables as median (IQR). Chi‐square and Mann–Whitney tests were used for comparisons of frequencies or medians respectively. Variables found be associated (*p* ≤ 0.05) with liver fibrosis and steatosis were entered into multivariate logistic regression models adjusted for age and gender. Metabolic syndrome and its individual parameters (central obesity, type 2 diabetes, dyslipidaemia and hypertension) were entered into different multivariate models to avoid the effect of collinearity. The severity of multicollinearity among variables entered in each multivariate model was quantified by the variance inflation factor (VIF) 22 and linear correlation by Spearman's correlation coefficient. Statistical analyses were performed using STATA statistical package for Windows (StataCorp LP, College Station, TX, USA).

## 3 Results

**Figure 1:**
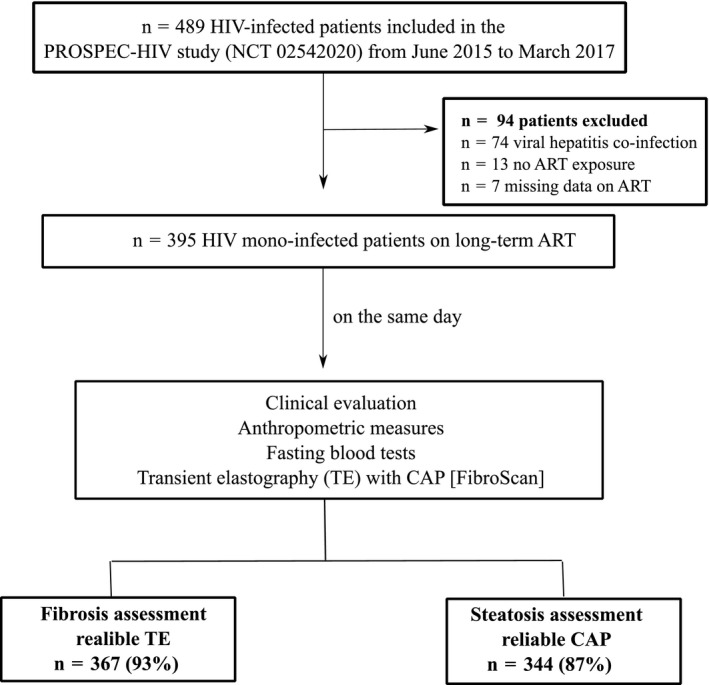
Study flow chart of patient's recruitment

A total of 489 patients with HIV infection were evaluated in the PROSPEC‐HIV study from June 2015 to March 2017. Patients were excluded due to viral hepatitis co‐infection (n = 74), no ART exposure (n = 13) or missing data on ART history (n = 7). Overall, 395 patients (60% female; median (IQR) age = 45 (35 to 52) years, body mass index = 25.7 (23.2 to 29.4) kg/m^2^, ALT levels = 30 (23 to 42) IU/L, 3% with ALT ≥ 3 × ULN, 15% with GGT levels ≥3 × ULN and 32% with metabolic syndrome) were included. The median (IQR) CD4+ T‐lymphocyte count was 667 (427 to 906) cells/mm^3^ and 20% (n = 80) of patients had a detectable HIV viral load >40 copies/mm^3^ (Table 1). In those patients with a detectable HIV activity, the mean of the HIV viral load was 550 copies/mm^3^ (range, 41 to 1,141,611)/2.55 log (range, 1.59 to 6.05). The duration of HIV infection and ART treatment were 10 (6 to 16) and 7 (4 to 14) years respectively. The majority of patients were actively receiving current ART drug classes of TDF‐backbone (78%) plus PI or INSTI (56%) and 43% of patients had AZT‐backbone as the most used historical ART‐backbone regimen (Table 2). LSM and CAP were unreliable in 28 patients (out of which 11 patients had no valid measurements) and 51 patients (out of which 13 patients had no valid measurements) respectively. Therefore, measurements from LSM and CAP for fibrosis and steatosis assessments were reliable for 93% (n = 367) and 87% (n = 344) respectively (Figure 1). Median (IQR) LSM and CAP were 5.3 (4.5 to 6.4) kPa and 230 (202 to 262) dB/m respectively. Liver fibrosis (LSM ≥ 8.0 kPa) was present in 9% (95% CI, 7 to 13) (n = 33) and liver steatosis (CAP ≥ 248 dB/m) in 35% (95% CI, 30 to 40) (n = 121) of patients (Table 2). The prevalence of advanced fibrosis (LSM ≥ 9.5 kPa) and cirrhosis (LSM ≥ 12.5 kPa) were 4.9% (95% CI, 3.1 to 7.7) and 1.4% (95% CI, 0.6 to 3.2) respectively. In addition, FIB‐4 < 1.45, APRI < 0.50 and NFS < −1.456 yielded 94%, 93% and 95% of negative predictive values for exclusion of liver fibrosis respectively.

**Table 1:** Characteristics of patients with HIV mono‐infection under long‐term ART

	All (n = 395)
Social and demographic characteristics	
Female gender^a^	236 (60)
Age, years^b^	45 [35 to 52]
Black/Brown race^a^	210 (53)
Probable route of HIV acquisition^a^	
Heterosexual women	191 (48)
Men who have sex with men	101 (26)
Heterosexual men	44 (11)
Other	22 (6)
Missing	37 (9)
Education level <8 years of study^a^	193 (49)
AUDIT score ≥8^a^	90 (23)
Former or current smoking^a^	184 (47)
Metabolic features	
BMI, kg/m²^b^	25.7 [23.2 to 29.4]
Central obesity (wc ≥ 90 cm in men and ≥80 cm in women)^a^	266 (68)
Type 2 diabetes^a^	37 (10)
Dyslipidaemia^a^	234 (61)
Hypertension^a^	118 (30)
Metabolic syndrome^a^	117 (32)
Biochemistry	
ALT, IU/L^b^	30 [23 to 42]
AST, IU/L^b^	26 [20 to 34]
GGT, IU/L^b^	46 [34 to 76]
Alkaline phosphatase, IU/L^b^	88 [69 to 107]
Total bilirubin, mg/dL^b^	0.43 [0.30 to 0.77]
Albumin, mg/dL^b^	3.9 [3.7 to 4.1]
Fasting glucose, mg/dL^b^	93 [87 to 100]
Triglycerides, mg/dL^b^	127 [87 to 178]
Total cholesterol, mg/dL^b^	185 [155 to 219]
LDL‐cholesterol, mg/dL^b^	112 [88 to 138]
HDL‐cholesterol, mg/dL^b^	42 [35 to 54]
HIV infection and ART history	
Duration of HIV infection, years^b^	10 [6 to 16]
CD4+ T‐lymphocyte count (cells/mm^3^)^b^	667 [427 to 906]
CD4+ T‐lymphocyte count <200 cells/mm^3^ a	17 (5)
Detectable HIV RNA viral load (>40 copies/mm^3^)^a^	80 (20)
Nadir CD4+ T‐lymphocyte count <100 cells/mm^3^)^a^	104 (26)
Duration of ART, years^b^	7 [4 to 14]
Current ART	
TDF‐backbone drugs^a^/AZT‐backbone drugs^a^	309 (78)/86 (22)
Core drugs treatment by NNRTI^a^/PI or INSTI^a^	175 (44)/220 (56)
Most used ART	
TDF‐backbone drugs^a^/AZT‐backbone drugs^a^	225 (57)/170 (43)
Core drugs treatment by NNRTI^a^/PI or INSTI^a^	197 (50)/198 (50)

Other probable routes for HIV infection: vertical transmission (n = 7), transfusion of blood products (n = 10), injected drug users (n = 3), occupational accident (n = 2).

ALT, alanine aminotransferase; ART, antiretroviral therapy; AST, aspartate aminotransferase; AUDIT, Alcohol Use Disorders Identification Test; AZT, zidovudine; BMI, body mass index; GGT, gamma‐glutamyltransferase; HDL, high‐density lipoprotein; INSTI, integrase strand transfer inhibitors; LDL, low‐density lipoprotein; NNRTI, non‐nucleoside reverse‐transcriptase inhibitors; PI, protease inhibitor, TDF, tenofovir; wc, waist circumference.

Data expressed as ^a^absolute (%) or ^b^median [IQR].

**Table 2:** Univariate and multivariate analyses for factors associated with liver fibrosis (LSM ≥ 8.0 kPa) in patients with HIV mono‐infection under long‐term ART

	Univariate analysis		Multivariate analysis	
	OR [95% CI]	*p* value	OR [95% CI]	*p* value
Social and demographic characteristics				
Female gender	1.10 [0.53 to 2.28]	0.805	1.04 [0.47 to 2.29]	0.927
Age (per 10 years)	1.78 [1.31 to 2.42]	<0.001	1.80 [1.27 to 2.55]	0.001
White race	1.44 [0.70 to 2.95]	0.323		
Education <8 years of study	1.73 [0.83 to 3.58]	0.143		
AUDIT score ≥8	0.57 [0.21 to 1.52]	0.258		
Former or current smoking	1.21 [0.59 to 2.48]	0.598		
Metabolic features				
Central obesity	1.21 [0.56 to 2.63]	0.632		
Type 2 diabetes	3.78 [1.48 to 9.68]	0.006	2.67 [0.96 to 7.46]	0.061
Dyslipidaemia	1.03 [0.48 to 2.20]	0.937		
Hypertension	2.19 [1.04 to 4.59]	0.038	1.30 [0.56 to 3.02]	0.535
Biochemistry				
ALT ≥ 1.5 ULN	3.40 [0.34 to 33.6]	0.296		
GGT ≥ 1.5 ULN	1.90 [0.77 to 4.65]	0.161		
Alkaline phosphatase ≥ 1.5 ULN	1.29 [0.15 to 10.7]	0.813		
HIV infection history				
Duration of HIV infection (per 10 years)	1.30 [0.79 to 2.16]	0.306		
CD4+ T‐lymphocyte count <200 cells/mm^3^	3.69 [1.12 to 12.2]	0.032	7.80 [2.09 to 29.09]	0.002
Detectable HIV RNA viral load (>40 copies/mm^3^)	0.98 [0.41 to 2.33]	0.957		
Nadir CD4+ T‐lymphocyte count <100 cells/mm^3^	1.42 [0.69 to 2.93]	0.338		
Duration of ART (per 10 years)	1.55 [0.89 to 2.69]	0.120		
Current treatment				
AZT‐backbone drugs (vs. TDF)	1.19 [0.51 to 2.74]	0.691		
PI or INSTI‐core drugs class (vs. NNRTI)	1.73 [0.81 to 3.68]	0.154		
Most used drugs during HIV infection				
AZT‐backbone drug class (vs. TDF)	1.49 [0.73 to 3.05]	0.275		
PI or INSTI‐core drugs class (vs. NNRTI)	1.44 [0.70 to 2.97]	0.322		

ALT, alanine aminotransferase; ART, antiretroviral therapy; AUDIT, Alcohol Use Disorders Identification Test; AZT, zidovudine; CI, confidence interval; GGT, gamma‐glutamyltransferase; INSTI, integrase strand transfer inhibitors; NNRTI, non‐nucleoside reverse‐transcriptase inhibitors; OR, odds ratio; PI, protease inhibitor, TDF, tenofovir; ULN, upper limit of normal.

### 3.1 Factors associated with liver fibrosis

Patients identified as having liver fibrosis were older (median (IQR) years; 52 (45 to 58) vs. 43 (34 to 51); *p* = 0.0001); were more likely to have a CD4+ T‐lymphocyte count <200 cells/mm^3^ (12% vs. 4%; *p* = 0.022), type 2 diabetes (21% vs. 7%; *p* = 0.003) and hypertension (44% vs. 26%; *p* = 0.035) when compared to those without fibrosis. The following factors were independently associated with fibrosis (odds ratio (OR) and CI): (i) older age (per 10 years; OR = 1.80 (95% CI, 1.27 to 2.55), *p* = 0.001) and low CD4+ T‐lymphocyte count (<200 cells/mm^3^; OR = 7.80 (95% CI 2.09 to 29.09), *p* = 0.002). In addition, those with type 2 diabetes had a trend towards the presence of liver fibrosis (OR = 2.67 (95% CI 0.96 to 7.46), *p* = 0.061). A significant multicollinearity was not observed in the fibrosis multivariate model (Table S1). We observed similar results using a higher LSM cutoff (≥9.5 kPa; n = 18; prevalence = 4.9% (95% CI, 3.1 to 7.7)): older age (per 10 years; OR = 3.91 (95% CI, 1.20 to 12.79), *p* = 0.024); low CD4+ T‐lymphocyte count (<200 cells/mm^3^; 5.15 (95% CI, 0.97 to 27.35), *p* = 0.054) and type 2 diabetes (yes vs. no; 3.99 (95% CI, 1.22 to 13.00), *p* = 0.022) were associated with fibrosis.

### 3.2 Factors associated with liver steatosis

Patients with liver steatosis were older (median (IQR) years; 48 (39 to 54) vs. 43 (33 to 51); *p* = 0.0001), a higher proportion were male (51% vs. 36%; *p* = 0.006) and self‐reported white race (58% vs. 43%; *p* = 0.007), had a longer duration (median (IQR)) of HIV infection (12 (7 to 17) vs. 9 (5 to 15) years; *p* = 0.0005), ART (9 (5 to 15) vs. 7 (3 to 12) years; *p* = 0.0024) and AZT‐backbone regimen (51 (0 to 136) vs. 13 (0 to 84) months; *p* = 0.0057), and a higher prevalence of metabolic syndrome (51% vs. 18%; *p* < 0.0001) compared to those without steatosis. The presence of central obesity (OR = 10.74 (4.40 to 26.20), *p* < 0.001), type 2 diabetes (OR = 9.74 (3.15 to 30.10), *p* < 0.001) and dyslipidaemia (OR = 2.61 (1.35 to 5.05), *p* = 0.003) were independently associated with liver steatosis. In addition, metabolic syndrome, used as a substitute for metabolic features, remained associated with steatosis in a multivariate model (OR = 4.28 (2.45 to 7.46), *p* < 0.001) (Table 3). However, a severe multicollinearity (VIF values) was observed in the multivariate model for duration of ART (VIF = 7.05), duration of HIV infection (VIF = 5.27) and cumulative years of use of AZT‐backbone drugs (VIF = 2.67). This collinearity was reinforced by the strong correlation (Spearman's rho, *p* value) between duration of ART with HIV infection duration (rho = 0.88, *p* < 0.001) and cumulative years of treatment using AZT‐backbone drugs (rho = 0.78, *p* < 0.001). In a sensitivity analysis, the duration of HIV infection (model A) (OR = 1.64 (1.05 to 2.54), *p* = 0.029), duration of ART (model B) (OR = 1.68 (1.03 to 2.72), *p* = 0.036) and AZT‐backbone regimen as most used drugs compared to TDF‐backbone (model C) (OR = 1.90 (1.07 to 3.38), *p* = 0.028) were associated with liver steatosis independently of metabolic features when these variables were entered separately in multivariate models (Table 4).

**Table 3:** Univariate and multivariate analyses for factors associated with liver steatosis (CAP ≥ 248 dB/m) in patients with HIV mono‐infection under long‐term ART

	Univariate analysis		Multivariate analysis			
			Model with metabolic features		Model with metabolic syndrome	
	OR [95% CI]	*p* value	OR [95% CI]	*p* value	OR [95% CI]	*p* value
Social and demographic characteristics						
Male gender	1.88 [1.20 to 2.94]	0.006	6.06 [2.85 to 12.88]	<0.001	1.94 [1.14 to 3.30]	0.015
Age (per 10 years)	1.48 [1.21 to 1.80]	<0.001	0.99 [0.75 to 1.32]	0.983	1.13 [0.89 to 1.44]	0.318
White race	1.85 [1.82 to 2.89]	0.007	1.45 [0.82 to 2.56]	0.204	1.64 [0.97 to 2.78]	0.067
Education <8 years of study	0.77 [0.49 to 1.19]	0.239				
AUDIT score ≥8	0.86 [0.51 to 1.45]	0.563				
Former or current smoking	1.05 [0.68 to 1.64]	0.818				
Metabolic features						
Central obesity	4.27 [2.39 to 7.62]	<0.001	10.74 [4.40 to 26.20]	<0.001	‐	‐
Type 2 diabetes	9.56 [3.52 to 25.97]	<0.001	9.74 [3.15 to 30.10]	<0.001	‐	‐
Dyslipidaemia	5.34 [0.02 to 9.43]	<0.001	2.61 [1.35 to 5.05]	0.004	‐	‐
Hypertension	1.96 [1.20 to 3.19]	0.007	0.67 [0.34 to 1.33]	0.253	‐	‐
Presence of metabolic syndrome	4.82 [2.87 to 8.07]	<0.001	‐	‐	4.28 [2.45 to 7.46]	<0.001
Biochemistry						
ALT ≥ 1.5 ULN	1.84 [0.26 to 13.22]	0.545				
GGT ≥ 1.5 ULN	1.43 [0.76 to 2.68]	0.264				
HIV infection and ART history						
Duration of HIV infection (per 10 years)	1.76 [1.27 to 2.43]	0.001	1.47 [0.59 to 3.62]	0.408	1.34 [0.57 to 3.12]	0.503
CD4+ T‐lymphocyte count <200 cells/mm^3^	0.54 [0.15 to 2.00]	0.355				
Detectable HIV RNA viral load (>40 copies/mm^3^)	0.53 [0.30 to 0.95]	0.034	0.58 [0.28 to 1.21]	0.145	0.59 [0.30 to 1.17]	0.133
Nadir CD4+ T‐lymphocyte count <100 cells/mm^3^	1.11 [0.71 to 1.73]	0.647				
Duration of ART (per 10 years)	1.72 [1.21 to 2.45]	0.003	0.98 [0.35 to 2.75]	0.966	1.17 [0.44 to 3.14]	0.758
Current treatment by AZT‐Backbone drugs (vs. TDF)	1.55 [0.92 to 2.62]	0.102				
Current treatment by PI or INSTI‐Core Drugs (vs. NNRTI)	0.93 [0.59 to 1.45]	0.737				
Most used AZT‐backbone drug treatment (vs. TDF)	2.03 [1.30 to 3.19]	0.002	1.62 [0.86 to 3.06]	0.135	1.29 [0.71 to 2.33]	0.410
Most used PI or INSTI‐Core Drugs treatment (vs. NNRTI)	0.91 [0.59 to 1.42]	0.685				

ALT, alanine aminotransferase; ART, antiretroviral therapy; AUDIT, Alcohol Use Disorders Identification Test; AZT, zidovudine; CI, confidence interval; GGT, gamma‐glutamyltransferase; INSTI, integrase strand transfer inhibitors; NNRTI, non‐nucleoside reverse‐transcriptase inhibitors; OR, odds ratio; PI, protease inhibitor, TDF, tenofovir; ULN, upper limit of normal.

**Table 4:** Multivariate analysis for factors associated with steatosis (CAP ≥ 248 dB/m) that entered variables duration of HIV infection (Model A), duration of ART (Model B) and cumulative use of AZT as a backbone drug (Model C) in different models

	Model A		Model B		Model C	
	Duration of HIV infection		Duration of ART		Cumulative use of AZT‐Backbone	
	OR [95% CI]	*p* value	OR [95% CI]	*p* value	OR [95% CI]	*p* value
Social and demographic characteristics						
Male gender	6.18 [2.93 to 13.06]	<0.001	6.36 [3.00 to 13.44]	<0.001	5.82 [2.77 to 12.21]	<0.001
Age (per 10 years)	1.01 [0.77 to 1.34]	0.929	1.02 [0.77 to 1.35]	0.920	1.07 [0.82 to 1.40]	0.610
White race	1.45 [0.82 to 2.55]	0.200	1.45 [0.82 to 2.55]	0.201	1.47 [0.83 to 2.59]	0.186
Metabolic features						
Central obesity	10.35 [4.29 to 25.00]	<0.001	10.72 [4.43 to 25.97]	<0.001	10.75 [4.44 to 25.99]	<0.001
Type 2 diabetes	9.44 [3.08 to 28.96]	<0.001	9.30 [3.05 to 28.39]	<0.001	9.42 [3.07 to 28.86]	<0.001
Dyslipidaemia	2.70 [1.40 to 5.20]	0.003	2.74 [1.42 to 5.30]	0.003	2.60 [1.35 to 5.03]	0.004
Hypertension	0.66 [0.34 to 1.30]	0.229	0.68 [0.35 to 1.34]	0.266	0.69 [0.35 to 1.35]	0.280
HIV infection and ART history						
Duration of HIV infection (per 10 years)	1.64 [1.05 to 2.54]	0.029				
Detectable HIV RNA viral load (>40 copies/mm^3^)	0.58 [0.28 to 1.20]	0.141	0.58 [0.28 to 1.20]	0.141	0.60 [0.29 to 1.24]	0.165
Duration of ART (per 10 years)			1.68 [1.03 to 2.72]	0.036		
AZT‐Backbone as the most used ART (vs. TDF)					1.90 [1.07 to 3.38]	0.028

ALT, alanine aminotransferase; ART, antiretroviral therapy; AZT, zidovudine; CI, confidence interval; INSTI, integrase strand transfer inhibitors; NNRTI, non‐nucleoside reverse‐transcriptase inhibitors; OR, odds ratio; PI, protease inhibitor, TDF, tenofovir.

## 4 Discussion

This study highlighted the burden of liver fibrosis and steatosis as assessed by TE in patients with HIV mono‐infection under long‐term ART. To the best of our knowledge, this is the first large‐scale study of this issue in people living with HIV in a resource‐limited setting. This study identified older age and low CD4+ T‐lymphocyte counts as being associated with liver fibrosis. In addition, standard metabolic factors and AZT, d4T, ddI or ddC as most used backbone drugs were related to hepatic steatosis.

Extensive variability remains regarding the prevalence of liver fibrosis and steatosis in patients with HIV mono‐infection. In a study of 62 individuals with HIV mono‐infection with persistently elevated aminotransferase levels having liver biopsies, Morse *et al*. reported a prevalence of steatosis and bridging fibrosis of up to 70% and 18% respectively 10. In a limited sample size (n =125) of consecutive patients with HIV infection followed in an European outpatient clinic, Lombardi *et al*. described prevalence rates of 55% for steatosis and 18% for fibrosis using abdominal ultrasound and LSM (≥7.4 kPa) respectively 13. In contrast, a study of 80 Asian individuals with HIV indicated lower prevalence rates of steatosis (29%) and fibrosis (14%) using magnetic resonance spectroscopy (MRS) and LSM (≥7.0 kPa) respectively 12. Our results are consistent with other large‐scale studies that defined liver steatosis and fibrosis by TE. Macias *et al*. reported 37% of steatosis (CAP ≥ 238 dB/m) in 326 consecutive patients with HIV mono‐infection followed in Spain 23. A study of 341 individuals with HIV mono‐infection in Germany demonstrated a prevalence of 10% fibrosis (LSM ≥ 7.2 kPa) 11. More recently, a large Canadian cohort (n = 541) reported similar prevalence of steatosis (36%) using CAP (≥248 dB/m) and higher rates of fibrosis (19%) using LSM (≥7.2 kPa) in people with HIV mono‐infection 14. Similar rates of liver fibrosis (LSM ≥ 7.2 kPa) were observed by the METAFIB study (n = 405) in France 15. The prevalence of steatosis and fibrosis in people living with HIV may coincide with the global obesity epidemic over the past decade 24. In the present study, the prevalence of liver fibrosis and steatosis was similar in hazard drinkers (AUDIT ≥ 8) compared to those without abusive alcohol intake.

Factors associated with liver fibrosis in patients with HIV mono‐infection remain controversial and the mechanisms of hepatic fibrogenesis are still unclear. In the present study, older age and CD4+ T‐lymphocyte count lower than 200 cells/mm^3^ were associated with fibrosis and type 2 diabetes showed a trend towards statistical significance, whereas duration of HIV infection, cumulative use of ART and metabolic syndrome were not. A low CD4+ T‐lymphocyte count may contribute to impaired stimulation of natural killer cells, which could result in reduced antifibrotic activity on hepatic stellate cells and consequently progression to liver fibrosis 25. We acknowledge that the relative low prevalence of patients with CD4+ T‐lymphocyte count <200 cells/mm^3^ and individuals with type 2 diabetes in the presence study sample should be taken into account when interpreting these results. In people living with HIV, central obesity and duration of HIV infection have been described by previous studies as key features for liver fibrosis 2, 11, 12, 14. Lemoine *et al*. reported higher LSM values in individuals with HIV infection with metabolic syndrome had compared to those without (mean (standard deviation), 6.3 (2.6) vs. 4.9 (1.5) kPa, *p* < 0.0001) 15. Additionally, a relationship between fibrosis and type 2 diabetes (OR = 5.47 (95% CI, 1.81 to 16.51)) was observed by Mohr *et al*. 11.

Liver fat accumulation is strongly correlated with metabolic factors and insulin resistance 3. However, data on patients with HIV mono‐infection are scarce and the impact of ART history on liver fat remains to be determined. Our results reinforced prior observations of metabolic features being strongly correlated with liver steatosis in people living with HIV 2, 14. In addition, we reported that the use of an AZT, ddI and/or d4T as the most used backbone regimen was associated with liver steatosis independent of metabolic features compared to TDF‐backbone (Table 4). This fact might be explained by mitochondrial toxicity caused by NRTIs that can be difficult to reverse 26. There is evidence‐based data for a central role of mitochondrial dysfunction in the complex pathophysiology of NAFLD 27 leading to oxidative stress, cellular apoptosis and adiponectin impairment 28. The World Health Organization (WHO) discourages the use of ddI and d4T in first‐line regimens, yet as recently as 2011 the WHO antiretroviral drug survey report found that 40% of patients in low‐ and middle‐income countries received d4T‐based regimens 29. In 2014, Franzeck *et al*. reported that up to 10% of patient in Africa were also receiving d4T‐based first‐line regimens 30. In the present study, the prevalence of steatosis was higher in patients with HIV suppression (38% (95% CI, 33 to 44)) compared to those with detectable HIV viral load (23% (95% CI, 15 to 33)). This higher prevalence of steatosis in virally suppressed patients might be associated with weight gain after ART initiation and/or non‐ART adherence in patients with detectable HIV, reinforcing the potential role of antiretroviral drugs on hepatic steatosis development. Regarding core drug classes, no significant association could be demonstrated between NNRTI‐ and PI‐ or INSTI‐based regimens and steatosis or fibrosis in this study, although recent data suggest that steatosis might be improved by switching from efavirenz to raltegravir 31. Recently, the Brazilian health authorities have recommended the use of dolutegravir for first‐line therapy 32. However, given the limited number of INSTI exposed (n = 35) patients in our study, we were unable to assess the impact of cumulative treatment by dolutegravir or raltegravir on liver steatosis.

The major limitations of this study were the cross‐sectional study design, the absence of HIV‐uninfected control group and the lack of liver biopsy or MRS to assess fibrosis and steatosis respectively. The cross‐sectional study design hinders the evaluation the dynamics of liver steatosis/fibrosis and the incidence of liver‐related complications. Liver biopsy has several limitations regarding limited feasibility, potential complications and sampling error 33 and this method might be an imperfect gold standard 34. In addition, for patients with HIV mono‐infection without evidence of liver disease, the risk of performing a liver biopsy may not be justified. The use of MRS is also not feasible in many settings as it is costly and not widely available 35. In people with HIV mono‐infection, the accuracy of TE for fibrosis 36 and steatosis assessment 37 were validated using liver biopsy and MRS as the reference respectively. In the present study, LSM ≥ 8.0 kPa was used as a cutoff indicative of clinically relevant fibrosis in patients with HIV mono‐infection following what has been suggested for the general population. However, LSM might be challenged by interobserver variability 38 and it can be overestimated due to non‐fasting status and presence of necroinflammatory activity, cholestasis, liver congestion and steatosis 39. In addition, fibrosis staging using the M probe can be limited by the presence of obesity and the XL probe can be an alternative in these patients. In order to minimize the risk of bias, all TE exams were performed by a single experienced operator in patients with an overnight fasting status. In addition, none of the patients had evidence of jaundice, decompensated heart failure and only a single patient had ALT levels ≥3 times ULN. In the present study, we chose to estimate simultaneously liver fibrosis and steatosis by TE in people living with HIV using validated cutoffs from the M probe and, as such, we have avoided the potential technical bias of using different probes. It is important to note that LSM (median (range)) was significantly higher in patients with steatosis by CAP compared to those without (5.6 (3.2 to 30.4) vs. 5.2 (2.8 to 21.3) kPa; *p* = 0.0182). A recent study reported that the diagnostic value of CAP for fatty liver might be unsatisfactory if the IQR of CAP ≥ 40 dB/m 40. If we had considered this threshold as a validity criterion for steatosis assessment, 82 additional patients (24%) should be excluded. In sensitivity analysis, however, we found a similar prevalence (n = 91/262; 35%) and similar factors associated with steatosis in patients with HIV mono‐infection using IQR of CAP < 40 dB/m as a reliability criteria (Table S2).

The strengths of this study include the fact that all procedures were performed on the same day, blood samples were analysed in a centralized laboratory and TE examinations were performed in a fasting status by a single experienced operator. Liver fibrosis was defined using a threshold of LSM suggestive of clinically relevant fibrosis. Our findings corroborate the use of low cutoffs for FIB‐4 and APRI to exclude liver fibrosis in resource‐limited settings. In addition, a major strength of the study is the availability of reliable data collected by trained investigators for INI/FIOCRUZ HIV cohort through the last 10 to 15 years that allowed the evaluation of the impact of HIV infection and cumulative use of different patterns of ART on fibrosis and steatosis.

## 5 Conclusions

In conclusion, the presence of hepatic fibrosis and/or steatosis represents a major health concern for patients with HIV mono‐infection. Non‐communicable diseases, such as obesity, type 2 diabetes and dyslipidaemia, can play a major role in the development of liver steatosis in individuals with HIV mono‐infection. In order to decrease the burden of hepatic events in people living with HIV, prevention and treatment of non‐communicable diseases need to be integrated into the existing HIV care services.

## Competing Interests

The authors have no conflict of interest to disclose related to this topic.

## Authors’ contributions

HP conceptualized and designed the study, collected, statistically analysed and interpreted the data, and drafted and critically reviewed the manuscript. SWC and CY interpreted the data and critically reviewed the manuscript. MM, NG, PS, CC and CA collected and interpreted the data, and critically reviewed the manuscript. PL interpreted the data and critically reviewed the manuscript. VGV and BG conceptualized and designed the study, supervised the study, interpreted the data and critically reviewed the manuscript.

## Supporting information


**Table S1**. Values of variance inflation factors (VIFs) of variables included in the multivariate model for predicting fibrosis and steatosis in patients with HIV mono‐infection
**Table S2**. Multivariate analysis for factors associated with liver steatosis (CAP ≥ 248 dB/m) in patients with HIV mono‐infection under long‐term ART using IQR of CAP < 40 dB/m as a validation criterion (n = 262)
**Table S3**. Multivariate analysis for factors associated with liver fibrosis (LSM ≥ 8.0 kPa) in patients with HIV mono‐infection including liver steatosis as a co‐variate
