# Supplementary material for: Predictive factors associated with liver fibrosis and steatosis by transient elastography in patients with HIV mono‐infection under long‐term combined antiretroviral therapy
Source: J Int AIDS Soc. 2018 Nov 5;21(11):e25201. doi: 10.1002/jia2.25201 (PMC6216177; doi:10.1002/jia2.25201)
Supplement: Supplementary file 1 — Table S1. Values of variance inflation factors (VIFs) of variables included in the multivariate model for predicting fibrosis and steatosis in patients with HIV mono‐infection Table S2. Multivariate analysis for factors associated with liver steatosis (CAP ≥ 248 dB/m) in patients with HIV mono‐infection under long‐term ART using IQR of CAP < 40 dB/m as a validation criterion (n = 262) Table S3. Multivariate analysis for factors associated with liver fibrosis (LSM ≥ 8.0 kPa) in patients with HIV mono‐infection including liver steatosis as a co‐variate [file JIA2-21-e25201-s001.docx]

**Supplementary Material**

**Predictive factors associated with liver fibrosis and steatosis by transient elastography in HIV mono-infected patients under long-term combined antiretroviral therapy**

**Authors Names**

Hugo Perazzo; Sandra W Cardoso; Carolyn Yanavich; Estevão P Nunes; Michelle Morata; Nathalia Gorni; Paula Simplicio da Silva; Claudia Cardoso; Cristiane Almeida; Paula Luz; Valdilea G Veloso; Beatriz Grinsztejn

| **Supplementary Tables** | **Page** |
| --- | --- |
| **Supplementary Table 1.** Values of variance inflation factors (VIFs) of variables included in the multivariate model for predicting fibrosis and steatosis in patients with HIV mono-infection | 1 |
| **Supplementary Table 2.** Multivariate analysis for factors associated with liver steatosis (CAP ≥ 248 dB/m) in patients with HIV mono-infection under long term ART using IQR of CAP < 40 dB/m as a validation criterion (n=262) | 2 |
| **Supplementary Table 3.** Multivariate analysis for factors associated with liver fibrosis (LSM ≥ 8.0 kPa) patients with HIV mono-infection including liver steatosis as a co-variate | 3 |
|  |  |

**Supplementary Table 1**. Values of variance inflation factors (VIFs) of variables included in the multivariate model for predicting fibrosis and steatosis in patients with HIV mono-infection

|  | **Fibrosis** | **Steatosis** | **Steatosis - Sensitivity analysis** | | |
| --- | --- | --- | --- | --- | --- |
|  | **Final Model** | **Final Model** | **Model A**  **Duration of HIV infection** | **Model B**  **Duration of ART** | **Model C**  **Cumulative use of AZT-Backbone** |
| Gender | 1.04 | 1.46 | 1.45 | 1.45 | 1.44 |
| Age (per 10 years) | 1.17 | 1.58 | 1.55 | 1.57 | 1.48 |
| White skin color |  | 1.08 | 1.08 | 1.08 | 1.08 |
| Central obesity |  | 1.55 | 1.55 | 1.54 | 1.54 |
| Type-2 diabetes | 1.07 | 1.10 | 1.09 | 1.09 | 1.10 |
| Dyslipidemia |  | 1.30 | 1.29 | 1.29 | 1.29 |
| Hypertension | 1.17 | 1.22 | 1.21 | 1.21 | 1.21 |
| Duration of HIV infection (per 10 years) |  | 5.27 | 1.26 |  |  |
| CD4 ^+^ T-lymphocyte count < 200 cells/mm^3^ | 1.02 |  |  |  |  |
| Detectable HIV RNA viral load (> 40 copies/mm^3^) |  | 1.03 | 1.03 | 1.03 | 1.03 |
| Duration of ART (per 10 years) |  | 7.05 |  | 1.24 |  |
| Cumulative use of AZT-Backbone drugs, years |  | 2.67 |  |  | 1.15 |
| Mean VIF of the model | 1.09 | 2.30 | 1.28 | 1.28 | 1.26 |

ART, antiretroviral therapy; AZT, zidovudine ; VIF, variance inflation factor

**Supplementary Table 2.** Multivariate analysis for factors associated with liver steatosis (CAP ≥ 248 dB/m) in patients with HIV mono-infection

under long term ART using IQR of CAP < 40 dB/m as a validation criterion (n=262)

|  | **Model A** | | **Model B** | | **Model C** | |
| --- | --- | --- | --- | --- | --- | --- |
|  | **Duration of HIV infection** | | **Duration of ART** | | **Cumulative use of AZT-Backbone** | |
|  | **OR [95%CI]** | **p value** | **OR [95%CI]** | **p value** | **OR [95%CI]** | **p value** |
| **Social and demographics characteristics** |  |  |  |  |  |  |
| Male gender | 7.01 [2.90-16.92] | < 0.001 | 7.30 [3.01-17.65] | < 0.001 | 6.66 [2.76-16.06] | < 0.001 |
| Age (per 10 years) | 0.95 [0.68-1.34] | 0.769 | 0.98 [0.69-1.38] | 0.886 | 1.05 [0.76-1.44] | 0.770 |
| White skin color | 1.52 [0.77-2.97] | 0.225 | 1.51 [0.77-2.94] | 0.229 | 1.51 [0.77-2.95] | 0.228 |
| **Metabolic features** |  |  |  |  |  |  |
| Central obesity | 10.71 [3.69-31.05] | < 0.001 | 10.86 [3.74-31.52] | < 0.001 | 10.41 [3.63-29.89] | < 0.001 |
| Type-2 diabetes | 7.59 [2.24-25.71] | 0.001 | 7.24 [2.17-24.11] | 0.001 | 6.56 [2.01-21.41] | 0.002 |
| Dyslipidemia | 3.66 [1.57-8.56] | 0.003 | 3.67 [1.57-8.58] | 0.003 | 3.56 [1.52-8.31] | 0.003 |
| Hypertension | 0.81 [0.37-1.74] | 0.582 | 0.82 [0.38-1.78] | 0.623 | 0.82 [0.38-1.78] | 0.625 |
| **HIV infection and ART history** |  |  |  |  |  |  |
| Duration of HIV infection (per 10 years) | 1.90 [1.12-3.24] | 0.018 |  |  |  |  |
| Positive HIV RNA viral load (> 40 copies/mm^3^) | 0.36 [0.15-0.86] | 0.021 | 0.37 [0.15-0.88] | 0.024 | 0.38 [0.16-0.90] | 0.029 |
| Duration of ART (per 10 years) |  |  | 1.80 [1.01-3.25] | 0.049 |  |  |
| AZT-Backbone as the most used ART (vs TDF) |  |  |  |  | 1.98 [1.01-3.91] | 0.048 |

ALT, alanine transaminase; ART, antiretroviral therapy; AUDIT, Alcohol Use Disorders Identification Test; AZT, zidovudine; BMI, body mass index; CI, confidence interval; GGT, gamma-glutamyltransferase; INSTI, integrase strand transfer inhibitors; NNRTI, non-nucleoside reverse transcriptase inhibitors; OR, odds ratio; PI, protease inhibitor, TDF, tenofovir (TDF); ULN, upper limit of normal

**Supplementary Table 3.** Multivariate analysis for factors associated with liver fibrosis (LSM ≥ 8.0 kPa) in patients with HIV mono-infection

including liver steatosis as a co-variate

|  | **Model A** | | **Model B** | |
| --- | --- | --- | --- | --- |
|  | **Steatosis (CAP ≥ 248 dB/m)** | | **Steatosis (CAP per dB/m)** | |
|  | **OR [95%CI]** | **p value** | **OR [95%CI]** | **p value** |
| **Including metabolic features** |  |  |  |  |
| Female gender | 1.02 [0.46-2.28] | 0.953 | 1.01 [0.45-2.25] | 0.983 |
| Age (per 10 years) | 1.79 [1.26-2.54] | 0.001 | 1.78 [1.25-2.53] | 0.001 |
| Type-2 diabetes | 2.60 [0.88-7.63] | 0.083 | 2.50 [0.86-7.26] | 0.091 |
| Hypertension | 1.31 [0.56-3.02] | 0.533 | 1.30 [0.56-3.00] | 0.539 |
| CD4 ^+^ T-lymphocyte count < 200 cells/mm^3^ | 7.79 [2.08-29.15] | 0.002 | 7.83 [2.09-29.43] | 0.002 |
| Presence of steatosis (CAP ≥ 248 dB/m) | 1.07 [0.46-2.49] | 0.875 | - | - |
| CAP measure (per dB/m) | - | - | 1.01 [0.99-1.02] | 0.665 |
| **Replacing metabolic features by steatosis** |  |  |  |  |
| Female gender | 1.03 [0.47-2.24] | 0.943 | 1.02 [0.46-2.22] | 0.969 |
| Age (per 10 years) | 1.85 [1.34-2.57] | < 0.001 | 1.84 [1.33-2.56] | < 0.001 |
| CD4 ^+^ T-lymphocyte count < 200 cells/mm^3^ | 6.01 [1.66-21.77] | 0.006 | 6.00 [1.65-21.89] | 0.007 |
| Presence of steatosis (CAP ≥ 248 dB/m) | 1.27 [0.58-2.77] | 0.553 | - | - |
| CAP measure (per dB/m) |  |  | 1.01 [0.99-1.01] | 0.452 |
